# Site-Directed Mutations in the C-Terminal Extension of Human αB-Crystallin Affect Chaperone Function and Block Amyloid Fibril Formation

**DOI:** 10.1371/journal.pone.0001046

**Published:** 2007-10-17

**Authors:** Teresa M. Treweek, Heath Ecroyd, Danielle M. Williams, Sarah Meehan, John A. Carver, Mark J. Walker

**Affiliations:** 1 Department of Chemistry, University of Wollongong, Wollongong, New South Wales, Australia; 2 Graduate School of Medicine, University of Wollongong, Wollongong, New South Wales, Australia; 3 School of Chemistry and Physics, The University of Adelaide, Adelaide, South Australia, Australia; 4 The University Chemical Laboratory, University of Cambridge, Cambridge, United Kingdom; 5 School of Biological Sciences, University of Wollongong, Wollongong, New South Wales, Australia; University of Arkansas, United States of America

## Abstract

**Background:**

Alzheimer's, Parkinson's and Creutzfeldt-Jakob disease are associated with inappropriate protein deposition and ordered amyloid fibril assembly. Molecular chaperones, including αB-crystallin, play a role in the prevention of protein deposition.

**Methodology/Principal Findings:**

A series of site-directed mutants of the human molecular chaperone, αB-crystallin, were constructed which focused on the flexible C-terminal extension of the protein. We investigated the structural role of this region as well as its role in the chaperone function of αB-crystallin under different types of protein aggregation, i.e. disordered amorphous aggregation and ordered amyloid fibril assembly. It was found that mutation of lysine and glutamic acid residues in the C-terminal extension of αB-crystallin resulted in proteins that had improved chaperone activity against amyloid fibril forming target proteins compared to the wild-type protein.

**Conclusions/Significance:**

Together, our results highlight the important role of the C-terminal region of αB-crystallin in regulating its secondary, tertiary and quaternary structure and conferring thermostability to the protein. The capacity to genetically modify αB-crystallin for improved ability to block amyloid fibril formation provides a platform for the future use of such engineered molecules in treatment of diseases caused by amyloid fibril formation.

## Introduction

The classic experiments of Anfinsen [1] on the folding of ribonuclease *in vitro* revealed that all the information required for folding of a polypeptide chain into its final native conformation is contained within the polypeptide chain itself. This is indeed evident with small proteins used for *in vitro* folding studies (e.g. barnase, 110 residues) which are able to refold to their active conformation in the absence of other proteins. In the case of large, multi-domain proteins encoded by long sequences, however, only a limited proportion achieve their native state unassisted [2]. Most are prevented from reaching this state by incorrect intermolecular interactions that occur when the protein is in a partially folded, intermediate state, whereby hydrophobic regions on their surface interact resulting in protein aggregation and precipitation. Thus, protein folding and unfolding are exquisitely regulated in the cell and involve molecular chaperone proteins that assist other proteins in adopting their correct, native state. The small heat shock proteins (sHsps) act in a chaperone manner by recognizing and stabilizing the intermediate states of target proteins, thereby preventing improper or incorrect folding that would otherwise result in protein misfolding, aggregation, precipitation and possibly disease [3], [4], [5], [6]. αB-Crystallin is a sHsp that is capable of interacting with a multitude of target proteins to prevent their aggregation and precipitation [7]. However, unlike the classical bacterial chaperonin GroEL, sHsps (including αB-crystallin) do not directly participate in refolding of the denatured proteins, except in the presence of another chaperone protein, e.g. Hsp70 [8]. sHsps act specifically with target proteins that are on their off-folding pathway [9]. αB-Crystallin is primarily found in the eye lens, where it associates with the closely related αA-crystallin, which has 57% sequence homology with αB-crystallin and shares the conserved ‘α-crystallin domain’ (reviewed in [10]), to form large hetero-oligomeric species. However, it is also constitutively expressed in many non-lenticular tissues, including the brain, lung and cardiac and skeletal muscle where it forms complexes with other sHsps [11]. As with other members of the sHsp family, the expression of αB-crystallin is dramatically up-regulated in response to stress and pathological conditions such as Alzheimer's, Parkinson's and Creutzfeldt-Jakob diseases [4], [12], [13], [14].

The sHsps form a structurally divergent protein family with members present in archaea, bacteria and eukarya [10]. Monomeric molecular masses of the sHsps range between 12 and 40 kDa, however, most form large oligomeric assemblies of 150–800 kDa. All members are characterized by the presence of a homologous sequence of 80–100 residues, referred to as the ‘α-crystallin’ domain [15]. This domain is preceded by an N-terminal domain, which is highly variable in size and sequence, and is followed by a C-terminal extension. Whilst the C-terminal extensions of family members share little sequence similarity, they have the common characteristics of being polar and having conformational flexibility similar to peptides of the same length [16]. In previous studies, we have identified by ^1^H Nuclear Magnetic Resonance (NMR) spectroscopy that Hsp25 and α-crystallin have short, flexible and solvent exposed C-terminal extensions, which protrude from the domain core of the molecule [16], [17], [18].

The primary role of the flexible C-terminal extension of α-crystallin is thought to be to act as a solubilizing agent for the relatively hydrophobic protein and the sHsp-protein complex formed by its chaperone action [19], [20]. It may also play a role in subunit interaction since the resolved crystal structures of plant [21] and archaeal [22] sHsps indicate that their C-terminal extensions wrap around the outer surface of the sHsp complex. A similar role for the C-terminal extension of yeast Hsp26 has also recently been reported [23]. During ageing, αB-crystallin undergoes truncation of four C-terminal residues which correlates with a reduction in the protective ability of the protein and an increase in cataract formation [24]. Enzymatic truncation of the C-terminus *in vitro* with calpain II or trypsin [25], [26] or immobilization of its flexibility through mutagenesis [27], results in a reduction in its chaperone ability against amorphously aggregating target proteins. Other site-directed modifications within the C-terminal domain have also been shown to decrease the chaperone activity of α-crystallin and other vertebrate sHsps against amorphously aggregating target species [27], [28], [29], [30]. Together these studies show that the C-terminal extension plays an important role in the chaperone function of sHsps. However, to date the role of individual amino acids in the C-terminal domain of the protein has not been explored.

The interaction of sHsps with fibril-forming proteins has been investigated (e.g. [31], [32]) and it has been shown that α-crystallin inhibits fibril formation by apolipoprotein C-II [33]. Moreover, wild-type αB-crystallin is able to suppress fibril formation by β-amyloid [34] and α-synuclein [35]. There is emerging evidence that conformational changes in αB-crystallin give rise to a more effective form of the chaperone. Phosphomimics of αB-crystallin with altered structure [36] and a chimeric form of α-crystallin (comprising αA- and αB-crystallin) have been shown to have enhanced chaperone activity against amyloid fibril formation compared to the wild-type protein [37].

In this study we have generated several mutants of αB-crystallin in which the mutation sites are located in the C-terminal region of the protein, in particular the flexible C-terminal extension. In order to examine the role of specific C-terminal residues in regulating the oligomeric structure and chaperone function of αB-crystallin we have used a variety of biophysical techniques. As well, the chaperone function of these mutants was compared with that of the wild-type protein under different types of protein aggregation, i.e. disordered amorphous aggregation and ordered amyloid fibril assembly. The latter is of particular importance due to its association with protein deposits found in diseases such as Alzheimer's, Parkinson's and Creutzfeldt-Jakob disease.

## Methods

All reagents used were of analytical grade unless otherwise specified. DEAE-Sephacel anion-exchange resin was obtained from Sigma Chemical Co. (St. Louis, U. S. A.). Sephacryl S-300HR size exclusion resin was obtained from GE Biosciences (Uppsala, Sweden). Phenylmethylsulphonylfluoride (PMSF), polyethylinimine (PEI), kanamycin sulphate, thioflavin T (ThT), bovine κ-casein and bovine pancreas insulin were obtained from Sigma. Prior to use, the κ-casein was reduced and carboxymethylated as described previously [38]. Bovine β_L_-crystallin was purified via size exclusion chromatography (SEC) using methods described elsewhere [39], [40] and subunit values involving β_L_-crystallin were calculated based on the mass of the most abundant component of bovine β_L_-crystallin, β_B2_, i.e. 26 kDa. The 18mer coiled-coil α-helical peptide described previously [41], with additional C-terminal tryptophan residue (ccβ-Trp) was synthesized by CS Bio Co. (San Carlos, CA, U. S. A.). Isopropanyl-β-D-thiogalactopyranoside (IPTG), 5-bromo-4-chloro-3-indolyl-β-galactopyranoside (X-Gal), biotechnology grade agarose and Wizard® SV DNA purification kits were all obtained from Promega (Madison, U. S. A). Tris base, D-(+) glucose, glycine, lysozyme, ampicillin sulphate and dithiothreitol (DTT) were purchased from Astral Scientific (Carringbah, Australia). Tryptone, agar and yeast extract were obtained from Oxoid (Heidelberg West, Australia). Snakeskin dialysis tubing was obtained from Pierce (Rockford, U. S. A). Restriction enzymes were obtained from Roche Biochemicals (Indianapolis, U. S. A), QuikChange® Site-Directed Mutagenesis kit was obtained from Stratagene® (La Jolla, U. S. A.), Big-Dye® Terminator ready reaction mix was obtained from Applied Biosystems (Foster City, U. S. A). Uranyl acetate was obtained from Agar Scientific (Essex, U. K.). The expression vector pET24d(+) (Novagen, Madison, U. S. A.) containing the gene for expression of human αB-crystallin was a gift from Prof. W. de Jong (University of Nijmegen, Netherlands).

### Site-directed mutagenesis of pET24d(+)-αB-crystallin

Site-directed mutagenesis was performed with the QuikChange® system (Stratagene®, La Jolla, U. S. A.) on a Corbett Research Cooled Palm 96 PCR machine (Corbett Research, Australia) as per the manufacturer's instructions with minor changes. All primers were synthesized by Sigma Genosys (Castle Hill, Australia) and 3% (v/v) Dimethylsulfoxide (DMSO) was added to mutagenic reactions in which strong secondary interactions were likely. Site-directed mutagenesis primers were as follows: I159A/I161A, 5′-GAGCGCACCGCTCCCGCCACCCGTGAAG-3′; R163STOP, 5′-ACCATTCCCATCACCTGAGAAGAGAAGCCTGCT-3′; E164A/E165A, 5′-AATCCCATCACCCGTGCAGCGAAGCCTGCTGTCACC-3′; K174A/K175A, 5′- GTCACCGCAGCCCCCGCGAAGTAGATGCCCTTTCTT-3′; K175L, 5′-ACCGCAGCCCCCAAGTTATAGATGCCCTTTCTT-3′.

Mutated codon/s are underlined. Site-directed mutagenesis was confirmed using DNA sequence analysis with the following primers: pET24d(+)-αB-crystallin forward 5′-GTCAACCTGGATGTGAAGCA-3′ and pET24d(+)-αB-crystallin reverse 5′-CATTCACTGGTGGGGAAACT–3′.

Plasmid DNA was routinely digested with *Nco* I and *Hind* III confirm the presence of αB-crystallin gene insert prior to DNA sequence analysis which was performed with Big-Dye Terminator Ready Reaction Mix (PE Biosystems, U. S. A.) on an ABI-PRISM 377 DNA sequencer (Applied Biosystems, U. S. A).

### Expression and purification of wild-type and mutant αB-crystallins

Wild-type αB-crystallin and its C-terminal mutants were expressed and purified as described previously [40] except that, following ion-exchange chromatography, the I159A/I161A αB-crystallin mutant was immediately dialyzed against 50 mM phosphate buffer (pH 8.0) to avoid its aggregation and both I159A/I161A and R163STOP αB-crystallin were also exhaustively dialyzed against 50 mM sodium phosphate buffer (pH 7.4) following size-exclusion chromatography in order to avoid the precipitation that occurred when they were dialyzed against milliQ-water. Protein concentrations were determined by spectrophotometric methods using a Cary 500 Scan UV-Vis-NIR spectrophotometer (Varian, Melbourne, Australia) using the extinction coefficient for αB-crystallin (*E* = 19,000) [40] and molecular masses of the various mutants from mass spectrometric analysis (Table 1).

**Table 1:** Molecular masses of recombinant proteins as determined by ESI-MS.

Protein	Predicted Mass (Da)	Actual Mass from ESI-MS (Da)
Wild-type αB-crystallin	20159.0^1^	20160.1^3^
E164A/E165A αB-crystallin	20043.0^2^	20043.0
I159A/I161A αB-crystallin	20074.9^2^	20074.4
K174A/K175A αB-crystallin	20044.9^2^	20044.8
K175L αB-crystallin	20144.0^2^	20143.9
R163STOP αB-crystallin	18753.2^2^	18752.0

1 As given by SwissProt protein database, accession number P02511;

2 Predicted masses were all calculated from average isotopic masses;

3 Nanospray ESI-MS was used for this sample.

### Electrospray Ionisation Mass Spectrometry (ESI-MS) and Nanoscale Electrospray Ionisation Mass Spectrometry (NanoESI-MS)

Samples for ESI-MS were lyophilized after dialysis against milliQ-water to remove any salts present (with the exception of the I159A/I161A and R163STOP αB-crystallin mutants, which were dialyzed against 100 mM ammonium acetate). These samples had a final protein concentration of 1 µM. Nano-ESI-MS and ESI-MS were performed on a Q-Tof™2 quadrupole orthogonal acceleration time-of-flight mass spectrometer (Micromass U. K., Manchester, U. K.) with a Nanoflow-Z™ interface. Resulting spectra were processed with MassLynx™ software.

### Intrinsic tryptophan fluorescence

All fluorescence studies were performed on a Hitachi F-4500 fluorescence spectrophotometer with a 3 mL quartz fluorescence cuvette. Wild-type and mutant αB-crystallins (10 µM) were prepared in 50 mM sodium phosphate buffer, pH 7.2 and tryptophan residues were excited at 295 nm and emission spectra recorded from 300 to 400 nm. Slit widths for excitation and emission were 5.0 and 2.5 nm, respectively.

### Circular Dichroism (CD) spectroscopy

CD spectra were acquired with a Jasco J-810 spectropolarimeter (Jasco, Victoria, Canada) with a Jasco circulating water bath at 25°C. Samples were prepared in pre-filtered 10 mM sodium phosphate buffer, pH 7.5. αB-Crystallin samples for both near-UV and far-UV spectra were approximately 1 mg/mL and were filtered before analysis using 0.22 µm syringe filters. Near-UV spectra were recorded from 240 to 320 nm in a 1 cm pathlength cell and far-UV spectra recorded from 195 to 240 nm in a 0.01 cm pathlength cell. In the near-UV and far-UV regions, spectra represent the average of 16 and 32 scans, respectively. Mean residue ellipticity was calculated from the protein samples absorbance at 280 nm, residue number and the molecular weight of each mutant and wild-type protein as determined by ESI-MS (Table 1).

### Size-exclusion HPLC (High Performance Liquid Chromatography)

Aggregate sizes of wild-type and mutant proteins were analyzed by size-exclusion chromatography on a Phenomenex® BioSep™ SEC S4000 column with an exclusion limit of 2,000,000 Daltons (Phenomenex®, Torrance, U. S. A). A GBC/ICI HPLC system was used (ICI, London, U. K.) with a mobile phase of 50 mM sodium phosphate buffer, pH 7.2, an LC 1150 pump and LC 1440 systems organizer. Samples and standards were prepared at 10 mg/mL in 50 mM sodium phosphate, pH 7.2, with 0.02% (w/v) NaN_3_, 2.5 mM EDTA and 0.1 M NaCl. A sample (10 µL) was injected onto the column and eluted at 0.5 mL/min. The column was calibrated under the same conditions using the following standards; blue dextran (2.0 MDa), bovine thyroglobulin (670 kDa), catalase (250 kDa), ovotransferrin (78 kDa), and bovine serum albumin (BSA) (67 kDa). Approximate mass ranges of the proteins were calculated from elution times at half-peak height.

### Thermostability studies

Wild-type and mutant αB-crystallins were prepared in 50 mM sodium phosphate buffer, pH 7.4. Protein (0.2 mg/mL) was heated in a 1 mL quartz cuvette from 35°C to 85°C in a Cary 500 Scan UV-Vis-NIR spectrophotometer equipped with a Cary temperature controller. Thermal ramping of 1°C/min was performed and protein precipitation in response to thermal stress was monitored by measuring light scattering at 360 nm.

### Chaperone activity assays

To test the relative activity of the chaperones, the aggregation and precipitation of the target proteins under stress conditions was monitored by either Thioflavin T (ThT) fluorescence or turbidity assay (see below). Assays were conducted in 96-microwell plates by incubating the target protein in the absence or presence of the wild-type and mutant αB-crystallins following initial shaking for 5–10 s. Light scattering at 340 nm was measured and recorded using a Fluostar Optima plate reader (BMG Labtechnologies, Melbourne, Australia). The relative change in light scattering at 340 nm for each sample is presented in the graphs. The change in light scattering in the buffer control was negligible for each assay. Bovine β_L_-crystallin (500 µg/mL) was incubated at 60°C in 50 mM phosphate buffer, pH 7.2. Bovine pancreas insulin (250 µg/mL) was incubated at 37°C in 50 mM phosphate buffer, pH 7.2 and the aggregation and precipitation of the insulin B-chain initiated by addition of dithiothreitol (DTT) to a final concentration of 10 mM.

The formation of amyloid fibrils by target proteins was monitored by an *in situ* ThT binding assay described previously [42], [43]. Briefly, reduced and carboxymethylated κ-casein; RCMκ-casein (500 µg/mL) and ccβ-Trp peptide (150 µg/mL) were incubated at 37°C in 50 mM phosphate buffer, pH 7.4 with 10 µM ThT, in the absence or presence of the αB-crystallin proteins. Microtitre plates were sealed to prevent evaporation and the fluorescence levels measured with a Fluostar Optima plate reader (BMG Labtechnologies) with a 440/490 nm excitation/emission filter set. The change in ThT fluorescence measured for each sample is presented. The change in ThT fluorescence in the absence of the target protein was negligible for each assay. Also, neither Bovine Serum Albumin (BSA) nor lysozyme had any effect on the increase in ThT fluorescence when used in these assays in place of αB-crystallin.

The relative chaperone ability of the αB-crystallin proteins was assessed at the end of each assay by calculating the percentage protection afforded by the chaperone using the formula:
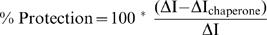
where ΔI and ΔI_chaperone_ represents the change in absorbance or ThT fluorescence for the target protein in the absence and presence of the chaperone respectively.

All experiments were replicated at least three times and the statistical significance of any differences observed between group means was determined by analysis of variance. Subsequent post-hoc testing of differences between group means was accomplished using the Tukey's test with *P*<0.05 considered significantly different.

## Results

Molecular masses and purities of human recombinant αB-crystallin proteins were assessed by ESI-MS (Table 1). The calculated masses of the wild-type (αB-WT) and mutant αB-crystallins were in excellent agreement with their predicted masses (max. 1.2 Da difference). Peaks in the mass spectra arising from contaminating proteins were minor (∼10% or less). Intrinsic tryptophan fluorescence of wild-type αB-crystallin produced a peak of fluorescence at 338.2 nm that arises from Trp9 and Trp60 (Table 2). The K174A/K175A, K175L and I159A/I161A mutants had red shifts in their wavelength maxima (λ_max_) compared to the wild-type protein (338.8, 340.0 and 341.0 nm, respectively). These data indicate that the tryptophan residues of these mutant proteins are more exposed than in the wild-type protein [36], [44], [45], [46], [47]. The E164A/E165A αB-crystallin mutant displayed the most significant change in tryptophan polarity with a blue shift in λ_max_ to 336.0 nm implying a conformational change in this mutant in which the tryptophan residues are buried in the hydrophobic core to a greater extent than in wild-type αB-crystallin. Similarly, the C-terminal truncation mutant (R163STOP) displayed a λ_max_ at a shorter wavelength (337.0 nm) than wild-type αB-crystallin (Table 2).

**Table 2:** Maximum tryptophan emission wavelengths for wild-type and mutant αB-crystallins.

Protein	λmax (nm)
Wild-type αB-crystallin	338.2
E164A/E165A αB-crystallin	336.0
I159A/I161A αB-crystallin	341.0
K174A/K175A αB-crystallin	338.8
K175L αB-crystallin	340.0
R163STOP αB-crystallin	337.0

**Figure 1:**
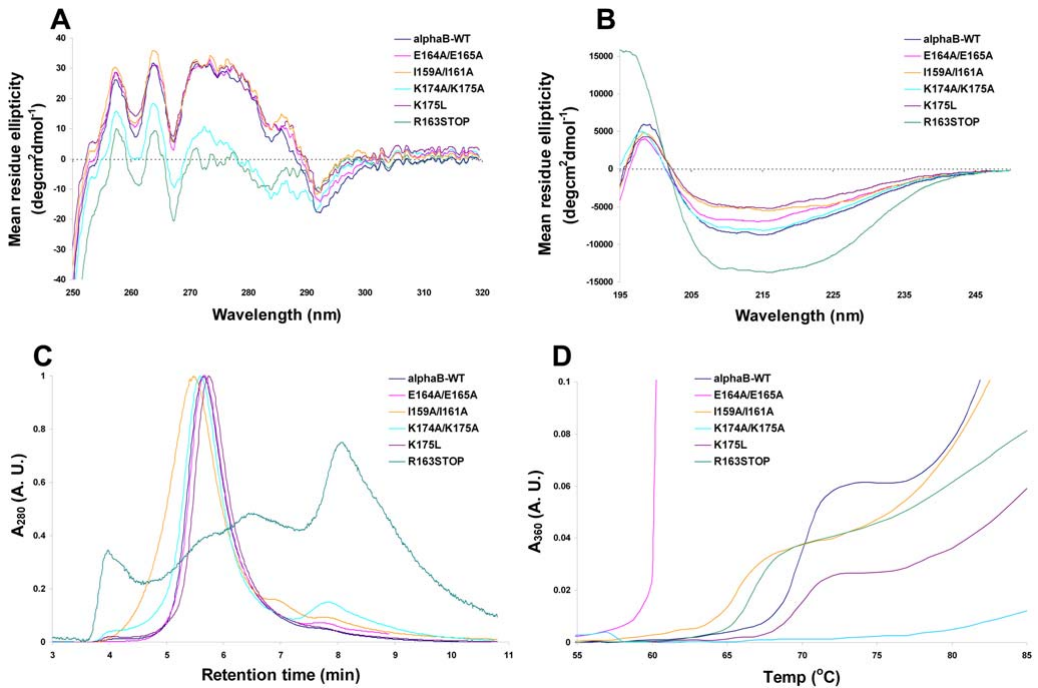
Biophysical characterization of wild-type and mutant αB-crystallin proteins. *A,* Near- and *B,* Far-UV CD spectra of wild-type and mutant αB-crystallin proteins. Spectra were acquired on a Jasco J-810 spectropolarimeter with 1 cm and 0.01 cm pathlength cells for the near- and far-UV regions, respectively. Protein concentrations were ∼1 mg/mL prepared in 10 mM sodium phosphate buffer, pH 7.4. *C,* Size-exclusion HPLC traces for wild-type and mutant αB-crystallin proteins in 50 mM sodium phosphate buffer, pH 7.2. Samples (10 µL of a 10 mg/mL solution) were injected onto the column and eluted at 0.5 mL/min. *D*, Thermostability profiles of αB-crystallin mutants. Protein samples were 0.2 mg/mL in 50 mM sodium phosphate buffer, pH 7.4. Thermal ramping was 1°C/min and light scattering at 360 nm was monitored as an indicator of protein precipitation.

The near-UV CD spectra of wild-type αB-crystallin exhibited a distinct minima at ∼267 nm and 292 nm and maxima at around 258, 264 and ∼273 nm (Fig. 1*A*) and was consistent with CD spectra presented previously [40], [48], [49]. The K175L, E164A/E165A and I159A/I161A mutants had very similar near-UV CD spectra to wild-type αB-crystallin indicating that their tertiary structures were not significantly altered by the mutation(s) (Fig. 1*A*). However, the spectra of K174A/K175A and R163STOP αB-crystallin showed a reduction in tertiary structure with a marked decrease in the maximum ellipticity at 273 nm. As summarized by Kelly [50] changes in this region of the spectrum are representative of the environment of the protein's tryptophan residues. Likewise, there is a reduction in the ellipticity of the bands corresponding to the phenylalanine residues (∼270 nm–250 nm). Alteration in the tryptophan environment of R163STOP and K174A/K175A is also reflected in the region of the spectrum from 280–292 nm. Taken together, these data are indicative of major tertiary structural changes induced by these mutations.

The far-UV CD spectrum of wild-type αB-crystallin (Fig. 1*B*) is characteristic of a predominantly *β*-sheet protein, with a minimum ellipticity at 213 nm. The majority of mutants had far-UV CD spectra almost identical to the wild-type protein with slight variations in ellipticity only (Fig. 1B). The R163STOP mutant showed significantly altered secondary structure compared to the wild-type and other mutant αB-crystallins. The change in overall shape of the spectrum, increased negative ellipticity (particularly in the region of ∼225 nm) and appearance of the strong positive band at ∼195 nm are all indicative of increased α-helical content. This would be consistent with the loss of the entire C-terminal extension, decreasing the relative amount of β-sheet content compared to α-helix. In the case of the K174A/K175A mutant, the loss of tertiary structure observed in the near-UV region with concurrent retention of secondary structure as seen in the far-UV region of the CD spectrum is indicative of a molten-globule like state similar to that observed for α-crystallin in the presence of a denaturant [51].

Most of the αB-crystallins eluted from the size exclusion column as relatively broad symmetrical peaks at early elution times indicating they exist as polydisperse high molecular mass oligomers (Fig. 1*C*). Wild-type αB-crystallin eluted in a single peak corresponding to an average molecular mass of 740 kDa and mass range of 420–980 kDa, consistent with previous studies [40], [52]. All of the mutants form oligomers that are more polydisperse than wild-type αB-crystallin (Fig. 1*C*). Both the E164A/E165A and K175L mutants form oligomers that have a slightly reduced average molecular mass (720 kDa and 670 kDa respectively) compared to the wild-type protein. In contrast, the average molecular mass of the K174A/K175A and I159A/I161A oligomers is higher than wild-type αB-crystallin (780 kDa and 880 kDa respectively). The average molecular masses of oligomers formed by the I159A/I161A mutant (up to 1.9 MDa) were found to be significantly larger than the wild-type when tested using an ANOVA with post-hoc analysis by Tukey's test (P<0.019). Upon visual inspection in can be seen that the ability of the R163STOP mutant to form large and consistently sized aggregates was profoundly impaired (Fig. 1*C*). Specifically, this mutant eluted in the form of a high molecular mass species in the void of the column (>2 MDa), at least two large molecular mass oligomers of 630 kDa and 310 kDa and a predominate peak corresponding to a species of less than 100 kDa.

When its thermostability was assessed, wild-type αB-crystallin began to precipitate from solution at 68°C and underwent a second phase of precipitation at 78°C (Fig. 1*D*). The E164A/E165A αB-crystallin mutant was much less thermostable than wild-type αB-crystallin and the other mutants used in this study as evidenced by its rapid, large-scale precipitation from solution at 60°C (Fig. 1*D*). The change in light scattering due to precipitation for the E164A/E165A mutant was more than 7-fold greater than for the other αB-crystallin proteins. The K175L mutant showed very similar thermostability to wild-type αB-crystallin with precipitation occurring in two phases at 68°C and 78°C. The R163STOP and I159A/I161A mutants were slightly less thermostable than wild-type αB-crystallin with onset of precipitation observed at 63°C and 65°C respectively. The K174A/K175A αB-crystallin showed enhanced thermostability compared to wild-type αB-crystallin, with only minimal precipitation evident at 85°C (Fig. 1*D*).

**Figure 2:**
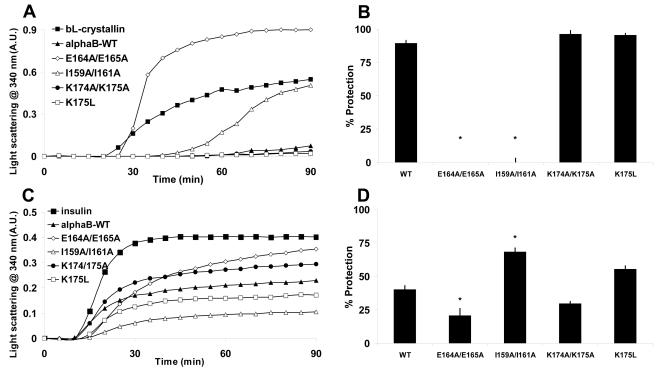
The chaperone ability of wild-type and C-terminal mutants of αB-crystallin to prevent amorphous aggregation. *A,* Heat-induced precipitation of β_L_-crystallin at 60°C and *C,* DTT-induced precipitation of insulin B-chain at 37°C, in the absence or presence of wild-type and mutant αB-crystallins at a 1.0:0.2 molar ratio of β_L_-crystallin: αB-crystallin and a 1.0:0.14 molar ratio of insulin: αB-crystallin. The change in light scattering at 340 nm for each sample is shown. Each assay was repeated four times and the data shown are representative. *B* and *D* show the percentage protection for each protein in the β_L_-crystallin and insulin B-chain aggregation assays respectively. The data in *B* and *D* represent the mean±standard error of the mean (SEM) of the 4 different experiments, * denotes a significant (p<0.05) difference in the mean compared to wild-type αB-crystallin.

The chaperone ability of the mutant αB-crystallin proteins was compared to wild-type αB-crystallin under a variety of conditions of induced target protein aggregation. The heat-induced amorphous aggregation of bovine β_L_-crystallin (a natural target of αB-crystallin in the lens), incubated in the absence of the chaperone, commenced after 20 min and the increase in light scattering due to protein precipitation reached a maximum after 80 min (Fig. 2*A*). Wild-type αB-crystallin was able to suppress the increase in light scattering in a concentration dependent manner, such that, at a 1.0:0.5 molar ratio of β_L_-crystallin: αB-crystallin, there was complete inhibition of protein precipitation (data not shown). At a 1.0:0.2 molar ratio of β_L_-crystallin:αB-crystallin the change in light scattering after 90 min was reduced by 90±2% (mean±SEM) (Fig. 2*A*). When the mutant αB-crystallin proteins were used at the same molar ratio (Fig. 2*B*), the E164A/E165A mutant was a significantly worse chaperone than the wild-type protein (p<0.01) and increased the amount of light scattering due to its own precipitation from solution at this temperature (see Fig. 2*A*). Whilst the I159A/I161A mutant delayed the onset of β_L_-crystallin's aggregation to 40 min, by the end of the assay the amount of light scattering in the presence of this mutant was similar to when β_L_-crystallin was incubated alone. The K174A/K175A and K175L proteins were effective chaperones at this sub-stoichiometric molar ratio to the target protein having activity similar to the wild-type protein.

Reduction of insulin with DTT induced amorphous aggregation of the B-chain after 10 min and the amount of light scattering due to protein precipitation reached a plateau after 45 min (Fig. 2*C*). Increasing the concentration of wild-type αB-crystallin prolonged the lag phase of aggregation and decreased the change in light scattering due to protein precipitation such that it was completely suppressed at a 1.0:0.4 molar ratio of insulin: αB-crystallin (data not shown). At a 1.0:0.14 molar ratio of insulin: αB-crystallin the lag phase of aggregation was the same as when insulin was incubated alone (10 min), but the rate of aggregation was decreased such that the amount of protein precipitation after 90 min was reduced by 40±3% (Fig. 2*C*). Comparing the chaperone activity of the αB-crystallin mutants to the wild-type protein at the same molar ratio showed that, whilst the E164A/E165A delayed the onset of aggregation to 15 min, by the end of the assay it was found to be a significantly worse chaperone (p<0.05) (Fig. 2*D*). In contrast, the I159A/I161A mutant was a significantly better chaperone (p<0.01), decreasing the amount of light scattering due to protein precipitation by 68±3% (Fig. 2*D*). Both the K174A/K175A and K175L mutants had similar chaperone ability as the wild-type protein in preventing the DTT-induced aggregation of the insulin B-chain.

We also used the R163STOP protein in both these amorphous aggregation assays to assess its relative chaperone ability compared to the wild-type protein, but due to its destabilized structure only a limited number of assays could be performed. The results of these assays suggested that the R163STOP protein is a much less efficient chaperone than wild-type αB-crystallin against both the heat-induced precipitation of β_L_-crystallin and DTT-induced precipitation of the insulin B-chain (data not shown).

**Figure 3:**
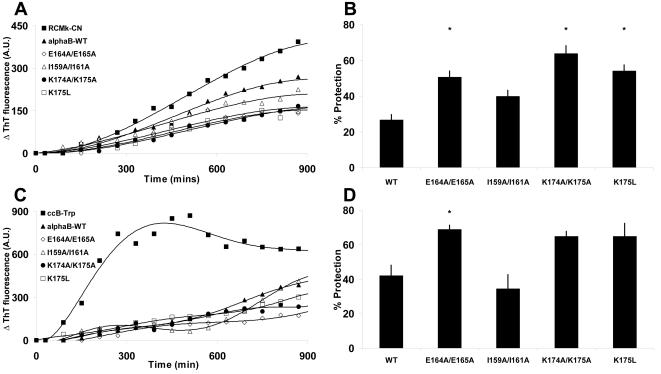
Inhibition of amyloid fibril formation by wild-type and C-terminal mutants of αB-crystallin. Thioflavin T (ThT) binding curves of *A,* RCMκ-casein and *C* ccβ-Trp incubated at 37°C in 50 mM phosphate buffer, pH 7.4 in the absence or presence of wild-type and mutant αB-crystallins. The chaperones were used at a 1.0:0.3 molar ratio of RCMκ-casein:αB-crystallin and a 1.0:0.05 molar ratio of ccβ-Trp:αB-crystallin respectively. The ThT fluorescence was monitored by *in situ* assay for 15 h and the change in ThT fluorescence of each sample is shown. Each assay was repeated four times and the data shown are representative. The data in *B* and *D* show the percentage protection for each protein in the RCMκ-casein and ccβ-Trp assay systems respectively. These data represent the mean±SEM of 4 different experiments, * denotes a significant (p<0.05) difference in the mean compared to wild-type αB-crystallin.

We employed two different models to examine the effect of C-terminal mutation on the ability of αB-crystallin to prevent amyloid fibril formation; reduced and carboxymethylated κ-casein (RCMκ-CN), an unstructured or “natively unfolded” protein [53]; and ccβ-Trp, a modified form of the ccβ peptide that exists in a helical coiled-coil configuration in its native state (Meehan, Ecroyd and Carver, unpublished data). We employed these two model systems since both form fibrils at physiological pH and temperature [36]. Fibril formation by RCMκ-CN, as monitored by an increase in ThT fluorescence, showed a gradual increase over the time course of the assay (Fig. 3*A*). The addition of αB-crystallin to the sample slowed the rate of fibril formation such that, at a 1.0:0.3 molar ratio of RCMκ-CN: αB-crystallin, the increase in ThT fluorescence was reduced by 26±3% (Fig. 3*B*). When the αB-crystallin mutants were used at the same concentration, the E164A/E165A, K174A/K175A and K175L mutants were more effective chaperones (p<0.01). The I159A/I161A mutant was found to have a similar level of chaperone ability against amyloid forming RCMκ-CN as the wild-type protein (Fig. 3*B*).

At 37°C, the increase in ThT fluorescence associated with fibril formation by ccβ-Trp was sigmoidal and included a short lag phase of 30 min followed by an increase in fluorescence which reached a plateau after 300 min (Fig. 3*C*). A 1.0:0.05 molar ratio of ccβ-Trp:αB-crystallin slowed the increase in ThT fluorescence and therefore, by inference, amyloid fibril formation. Whilst the overall trends in the relative chaperone ability of the wild-type and mutant proteins were consistent with this assay, the absolute percentage protection afforded by the chaperones was found to vary between assays. The E164A/E165A αB-crystallin mutant was more effective (p<0.01) at inhibiting the increase in ThT fluorescence compared to the wild-type protein. The I159A/I161A αB-crystallin mutant was found to be a very effective chaperone for the first 600 min of the assay, but the increase in ThT fluorescence increased significantly at this point such that, by 900 min, it afforded similar levels of protection against amyloid fibril formation as wild-type αB-crystallin (Fig. 3*D*). The K175L and K174A/K175A mutants had similar levels of chaperone ability as the wild-type protein.

## Discussion

We have successfully produced and expressed a number of mutants in the C-terminal region of αB-crystallin in order to investigate the role of this region, and individual residues within it, on the structure and chaperone function of this sHsp. In particular, we have targeted key residues within the C-terminal extension of the protein since it is thought to play an important role in the chaperone action of other sHsps [19], [20], [29], [54]. Overall, our studies are consistent with previous work showing that a highly flexible C-terminal extension in sHsps is important in the overall structure of the chaperone protein. Significantly, we found that certain residues in αB-crystallin play a key role in regulating its chaperone activity towards particular types of protein aggregation (i.e. ordered or disordered) and, therefore, these results may provide a foundation for future studies aimed at preventing diseases associated with protein aggregation.

Our results have also shown that the C-terminal extension plays a significant role in the oligomerization of αB-crystallin. Altered oligomerization properties and increased polydispersity have been shown to correlate with altered chaperone action of the α-crystallins [52], [55]. Each of the αB-crystallin mutants in this study exhibited perturbed oligomeric association such that they had broader molecular mass ranges than the wild-type protein. The most dramatic changes were for the I159A/I161A and R163STOP mutants. The I159A/I161A mutant eluted earlier and formed aggregates that were much more polydisperse than the wild-type protein, i.e. approximately 1.8 MDa to 490 kDa in mass (with an average mass of 880 kDa). The I-X-I/V motif is well conserved among sHsps, and in wheat Hsp 16.9 [21] and archael Hsp16.5 [22], plays a critical role in oligomeric assembly. Indeed, elimination of this motif from bacterial HspH leads to the complete abrogation of the ability of the protein to oligomerise and chaperone amorphously aggregating citrate synthase [56]. Our data support a role for this motif in regulating the oligomerization of αB-crystallin (see Fig. 1). The altered fluorescence spectra of the various αB-crystallin mutants compared to the wild-type protein are particularly interesting since the tryptophan residues in αB-crystallin are located in the N-terminal domain (Trp9 and Trp60). Our results indicate that in the case of K175L, K174A/K175A and I159A/I161A αB-crystallin, the mutations introduced resulted in conformational changes which led to the N-terminal tryptophan residues becoming more exposed to solvent. In contrast, the tryptophan fluorescence data of the E164A/E165A mutant show that the conformational changes induced by the loss of the two charged glutamic acid residues resulted in the N-terminal region becoming buried to a greater extent. A similar change was observed with the truncation of the C-terminal extension in R163STOP αB-crystallin. As shown by size-exclusion chromatography, the ability of this mutant to form regular multimeric complexes was also profoundly impaired. The removal of the highly flexible C-terminal extension led to the formation of heterodisperse αB-crystallin multimers ranging from more than 100 subunits to less than 5 in size. These results are consistent with studies of αA-crystallin showing that removal of residues from the C-terminal extension of the protein alters its oligomeric distribution and decreases its subunit exchange rate [55], [57]. Together, the results show that the C-terminal extensions of both αA- and αB-crystallin are not only important in maintaining the solubility of α-crystallin and its complex with target proteins but also in regulating the oligomerization of α-crystallin subunits into multimeric complexes. The C-terminal extension was one region identified as being a subunit-subunit interaction site in human αB-crystallin [58]. It has been proposed that the flexibility and polarity of the C-terminal extension of Hsps plays an important role in maintaining the spacing between adjacent complexes, which would otherwise be prone to aggregation via hydrophobic interactions [27]. Our results are consistent with this since disruption of this spacing mechanism would be expected to give rise to greater polydispersity, as is observed by size exclusion HPLC (see Fig. 1*C*).

Truncation of the entire C-terminal extension (R163STOP) of αB-crystallin also significantly altered the protein's secondary and tertiary structure (Fig. 1A and B). Together with the tryptophan fluorescence data, these results indicate that the C-terminal extension, although unstructured, plays a significant role in stabilizing the structure of the entire protein. A similar effect was observed in a C-terminally truncated mutant of Hsp25 (Hsp25ΔC18) where a loss of secondary and tertiary structure, compared to the wild-type protein, was evidenced by far-UV CD spectroscopy and 2D NMR spectroscopy [20]. The poor chaperone activity of R163STOP αB-crystallin against amorphously aggregating target proteins is in keeping with previous findings on deletion mutants of Hsps, i.e. Hsp25 [20], Hsp16.2 from *C. elegans* [54] and αA-crystallin [29].

Compared to the wild-type protein, the I159A/I161A αB-crystallin mutant was a significantly worse chaperone against heat-induced amorphously aggregating β_L_-crystallin, but was a significantly better chaperone against the reduction-induced amorphous aggregation of insulin (Fig. 2). These results are consistent with previous studies on an I159/161G mutant of αB-crystallin which exhibited enhanced chaperone ability under reduction stress [59]. In the present study, this mutant had similar chaperone ability against amyloid fibril forming targets to the wild-type protein highlighting the role of particular residues within the protein in regulating chaperone action of αB-crystallin during different stress conditions. For example, the increase in chaperone activity of I159A/I161A compared to wild-type αB-crystallin in the reduction-induced amorphous aggregation assay conducted at physiological temperatures may be due to the substitution of the two bulky, hydrophobic isoleucine residues in the I-X-I motif with more compact alanine residues. This in turn is likely to provide the C-terminal extension with greater flexibility and hence, increase the capacity of αB-crystallin to bind to some target proteins. Another possibility is that the substitution of these residues gives rise to a conformational change which further exposes putative chaperone binding site(s) [21]. Interestingly, however, these residues were not found to play a significant role in the chaperone action of αB-crystallin against amyloid fibril forming target proteins as there was no significant difference in the chaperone action of this mutant and the wild-type protein. Together, these data imply that the highly conserved I-X-I motif in sHsps regulates exposure of the hydrophobic groove of the α-crystallin domain of the protein, thereby controlling chaperone action with amorphously aggregating target proteins.

The increased thermostability of the K174A/K175A is consistent with the results of Liao *et al.* who showed that removal of the C-terminal lysine residue in porcine αB-crystallin led to a mutant with a higher thermostability than the wild-type protein [60]. In contrast, replacement of the ultimate lysine residue of the C-terminal extension with a leucine residue did not affect the protein's thermostability to any significant extent [60]. Significantly, our results show that the removal of these lysine residues does not compromise the chaperone activity of the protein against amorphously aggregating target proteins and increased its chaperone ability against aggregating RCMκ-CN. Thus, these data suggest that αB-crystallin does not require the two electropositive C-terminal lysine residues in order to interact with aggregating target proteins.

The double glutamic acid αB-crystallin mutant (E164A/E165A) showed greatly decreased thermostability and as a result was ineffective as a chaperone in the heat stress assay. Whilst the E164A/E165A mutant was able to delay the onset of aggregation of reduced insulin, by the end of the assay it was found to be a significantly worse chaperone than wild-type αB-crystallin. This probably reflects the relative instability of the complex formed between the insulin B-chain and E164A/E165A mutant protein. Interestingly, E164A/E165A αB-crystallin was a significantly better chaperone against the two amyloid fibril forming target proteins used in this study. Thus, E164 and E165, which are conserved between αA-and αB-crystallin, may play a role in regulating chaperone interaction during different types of target protein aggregation. The thermal instability of E164A/E165A αB-crystallin may be attributable to the removal of hydrophilicity that normally facilitates hydrophobic interactions within the protein, since hydrophobic interactions increase with increasing temperature. By analogy with hyperthermophilic proteins, non-covalent intermolecular interactions e.g. electrostatic and hydrogen bonding [61], [62] are facilitated by a high proportion of charged amino acids that act to stabilise the protein at high temperatures, via the formation of hydrogen bonds and salt bridges [62], [63], [64]. This solubilising role is likely to be important both for the αB-crystallin aggregate and the complex it forms with target proteins. Furthermore, due to electrostatic repulsion, these two glutamic acid residues may also function to separate protein subunits by blocking hydrophobic interactions and thereby regulating aggregate formation under heat stress.

Our results therefore highlight the important role of the C-terminal region, and in particular the flexible C-terminal extension, in the structure and chaperone function of αB-crystallin. Of particular interest, specific amino acid residues within the C-terminal region regulate its chaperone action against different types of protein aggregation (i.e. ordered and disordered). The mechanism by which αB-crystallin inhibits fibril growth is not known but it may do so by interacting with and binding pre-fibrillar aggregates in a soluble complex as it does with amorphously aggregating proteins [36]. In the case of α-synuclein, a component of Lewy bodies in Parkinson's disease, αB-crystallin is thought to interact with the fibril-forming species at an early stage of its aggregation pathway to reduce the nucleation rate thereby inhibiting fibril formation at the nucleation phase [35]. α-Crystallin also interacts with early amyloidogenic precursors of apolipoprotein C-II to inhibit nucleation [33]. Studies on the Alzheimer's disease protein, Aβ peptide, showed that αB-crystallin affected the elongation phase of fibril formation resulting in shorter, non-regular fibrils [32]. More recent studies on the same protein have shown that the chaperone preferentially binds to the fibril nucleus preventing propagation of fibrillar species [37].

It has been suggested that αB-crystallin operates more effectively as a chaperone against some target proteins than others as a result of the differing affinities of the chaperone for the various intermediately folded forms of its target [65], [66]. These observations are also consistent with αB-crystallin possessing more than one chaperone binding site, e.g. for amorphously aggregating and/or amyloid forming proteins. The occurrence of numerous binding sites on proteins which perform diverse functions has been well documented. Hsp90 has been shown to possess two independent binding sites with differential specificity [67] and the *E. coli* chaperone SecB has multiple binding sites which are both hydrophobic and hydrophilic in nature [68]. As discussed by Haslbeck *et al.*, the lack of conservation of residues amongst sHsps makes it difficult to locate potential target protein binding sites [69]. It is possible, therefore, that variable regions of sequence are involved in binding which would help to explain why sHsps such as αB-crystallin have such different target proteins affinities [69]. It may also support the hypothesis that multiple target-protein binding sites exist in the αB-crystallin sequence, which are affected to a greater or lesser degree by conformational change.

Studies have shown that contacts between the I-X-I motif in the C-terminal region and a hydrophobic grove in the αB-crystallin domain on an adjacent monomer are critical for proper oligomeric assembly in a plant sHsp [21]. It is possible; therefore, that irregular oligomeric assembly of mutants such as E164A/E165A and I159A/I161A αB-crystallin which form oligomers that are smaller and larger, respectively, than wild-type αB-crystallin also alters target protein binding at various sites. In the case of the E164A/E165A mutant, for example, removal of the pair of charged residues induced a conformational change which decreased the protein's stability under heat stress, altered oligomerization and unfavorably affected the binding of amorphously aggregating proteins. It is unclear at this stage whether the reduced binding of such disordered aggregating species occurs via obstruction or destabilization of such sites. In doing so, however, it is feasible that the same conformational changes may have exposed or stabilized another binding site on the molecule for fibril forming proteins.

The observation that genetically modified αB-crystallin, in comparison to the wild-type protein, demonstrates superior chaperone ability to suppress the formation of amyloid fibrils is a step in the potential development of treatments for diseases associated with amyloid formation and deposition. Future studies using *in vivo* models of amyloid fibril formation will elucidate the potential of modified αB-crystallin (i.e. E164A/E165A) to serve as therapeutic agents.
